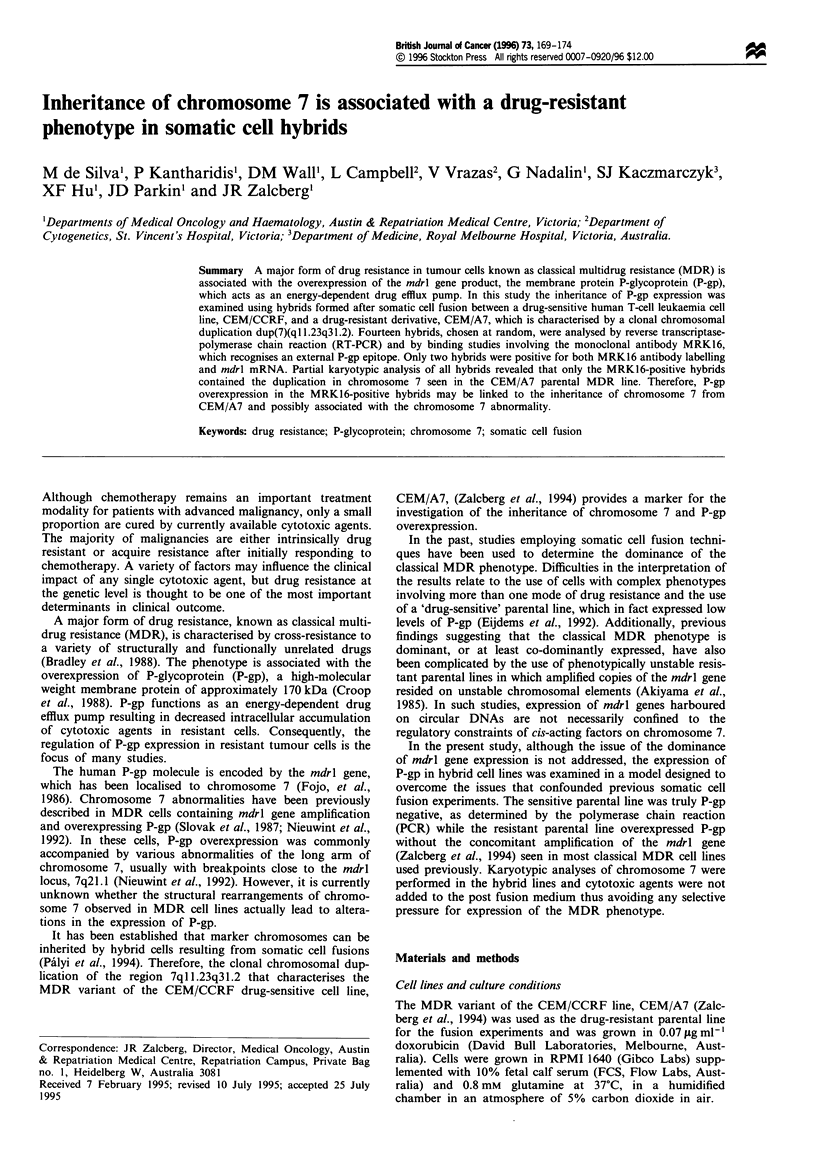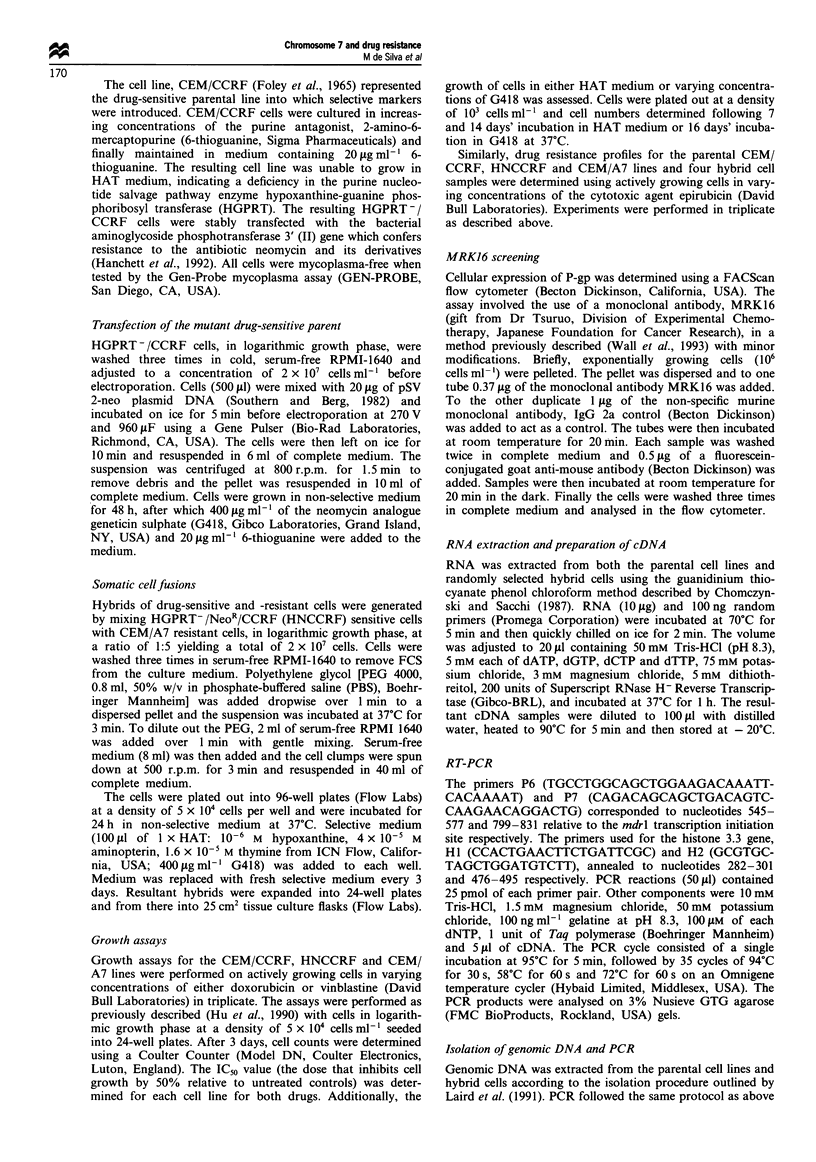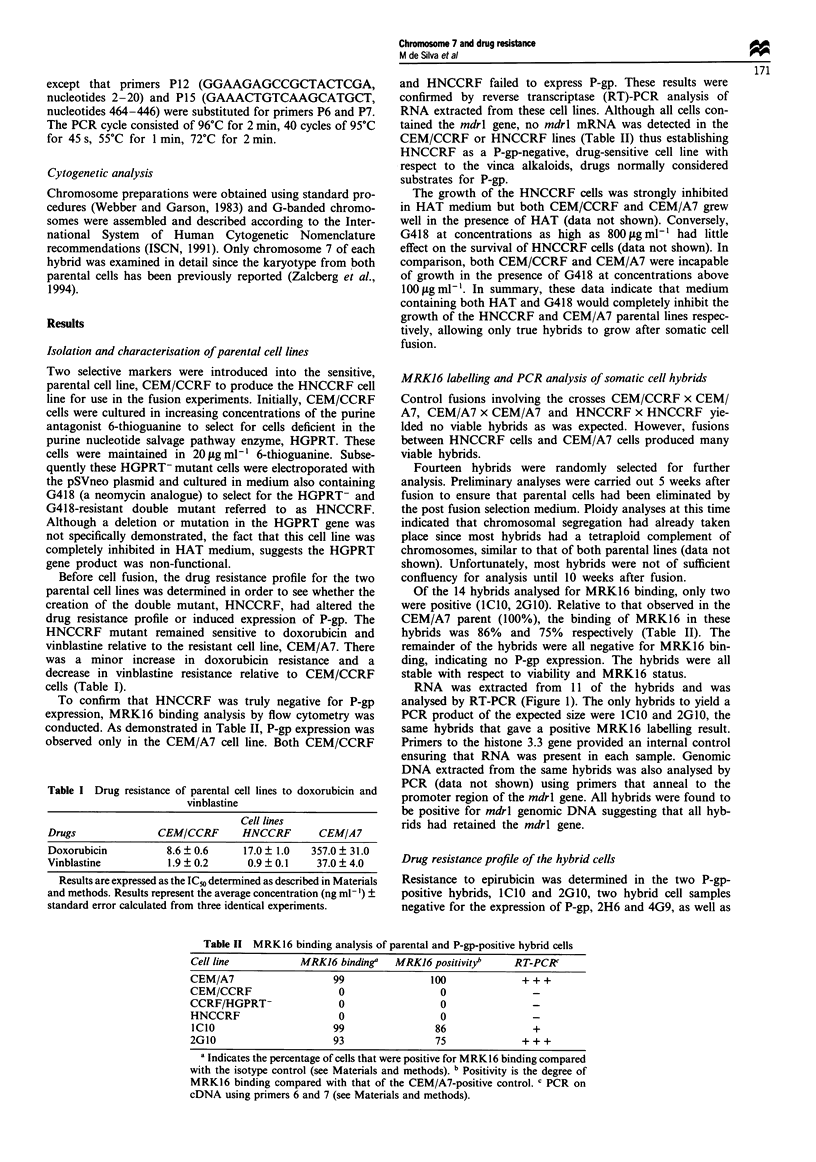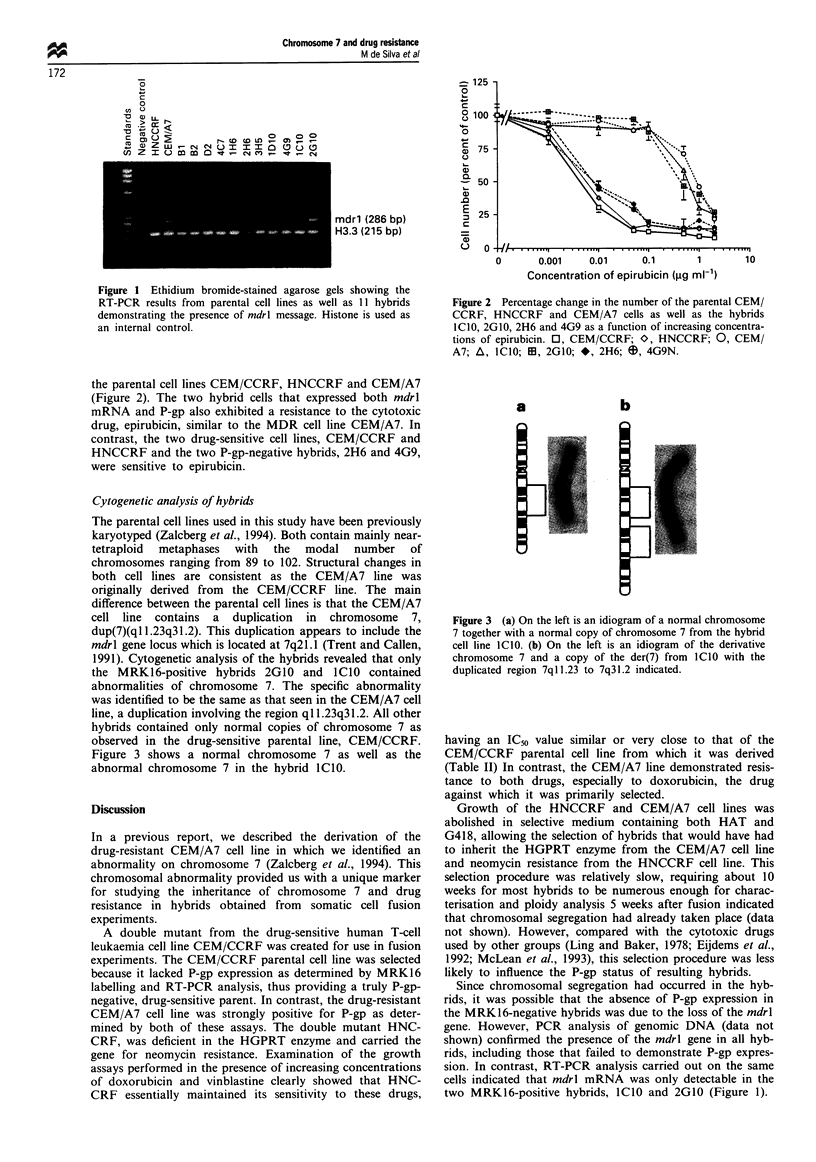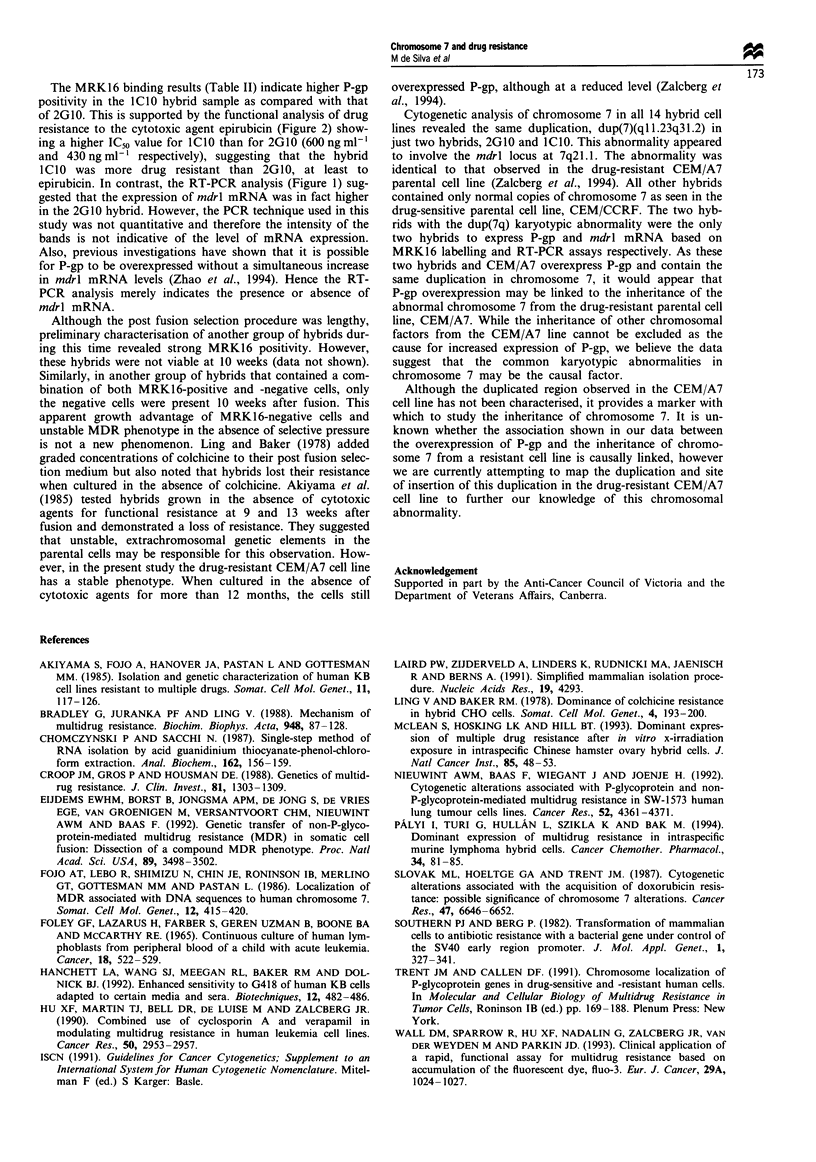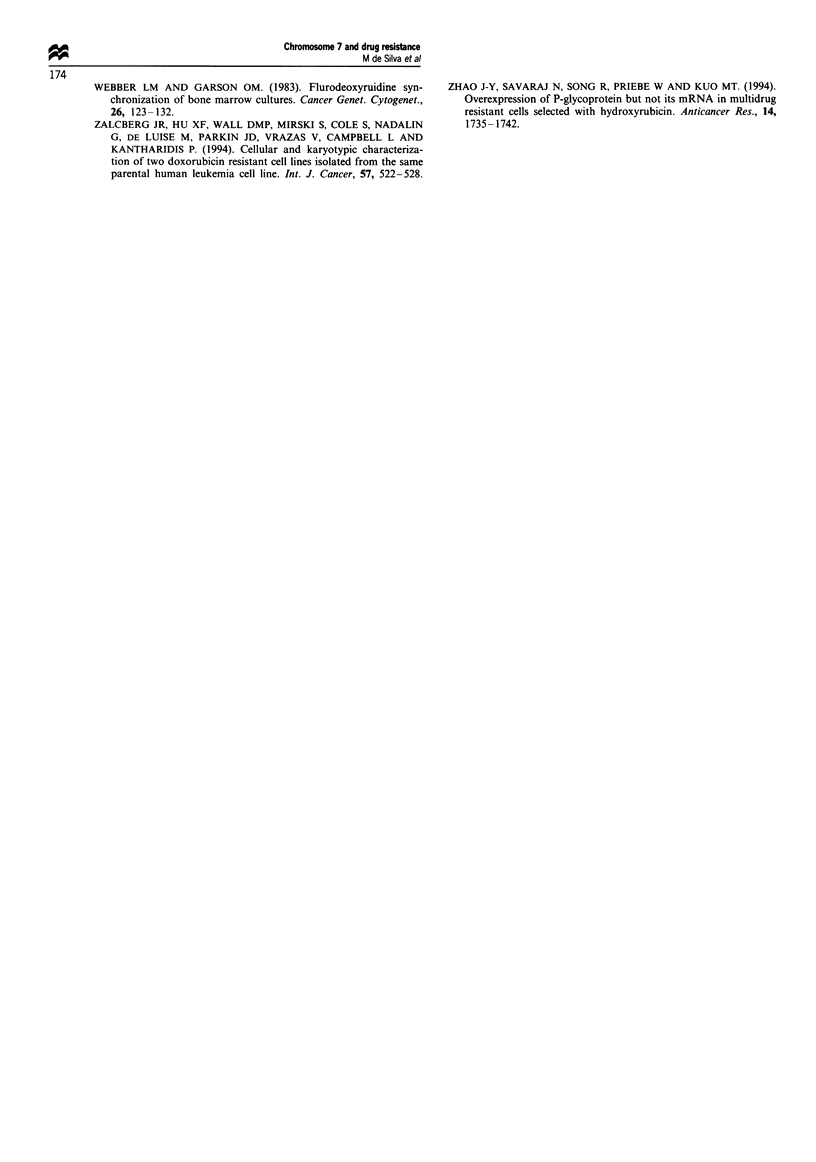# Inheritance of chromosome 7 is associated with a drug-resistant phenotype in somatic cell hybrids.

**DOI:** 10.1038/bjc.1996.31

**Published:** 1996-01

**Authors:** M. de Silva, P. Kantharidis, D. M. Wall, L. Campbell, V. Vrazas, G. Nadalin, S. J. Kaczmarczyk, X. F. Hu, J. D. Parkin, J. R. Zalcberg

**Affiliations:** Department of Medical Oncology, Austin & Repatriation Medical Centre, Victoria, Australia.

## Abstract

**Images:**


British Journal of Cancer (1996) 73, 169–174
© 1996 Stockton Press All rights reserved 0007-0920/96 $12.00

### Inheritance of chromosome 7 is associated with a drug-resistant phenotype in somatic cell hybrids

M de Silva[1], P Kantharidis[1], DM Wall[1], L Campbell[2], V Vrazas[2], G Nadalin[1], SJ Kaczmarczyk[3], XF Hu[1], JD Parkin[1] and JR Zalcberg[1]

[1]*Departments of Medical Oncology and Haematology, Austin & Repatriation Medical Centre, Victoria;* [2]*Department of Cytogenetics, St. Vincent's Hospital, Victoria;* [3]*Department of Medicine, Royal Melbourne Hospital, Victoria, Australia.*

**Summary** A major form of drug resistance in tumour cells known as classical multidrug resistance (MDR) is associated with the overexpression of the *mdr*1 gene product, the membrane protein P-glycoprotein (P-gp), which acts as an energy-dependent drug efflux pump. In this study the inheritance of P-gp expression was examined using hybrids formed after somatic cell fusion between a drug-sensitive human T-cell leukaemia cell line, CEM/CCRF, and a drug-resistant derivative, CEM/A7, which is characterised by a clonal chromosomal duplication dup(7)(q11.23q31.2). Fourteen hybrids, chosen at random, were analysed by reverse transcriptase-polymerase chain reaction (RT-PCR) and by binding studies involving the monoclonal antibody MRK16, which recognises an external P-gp epitope. Only two hybrids were positive for both MRK16 antibody labelling and *mdr*1 mRNA. Partial karyotypic analysis of all hybrids revealed that only the MRK16-positive hybrids contained the duplication in chromosome 7 seen in the CEM/A7 parental MDR line. Therefore, P-gp overexpression in the MRK16-positive hybrids may be linked to the inheritance of chromosome 7 from CEM/A7 and possibly associated with the chromosome 7 abnormality.

**Keywords:** drug resistance; P-glycoprotein; chromosome 7; somatic cell fusion

Although chemotherapy remains an important treatment modality for patients with advanced malignancy, only a small proportion are cured by currently available cytotoxic agents. The majority of malignancies are either intrinsically drug resistant or acquire resistance after initially responding to chemotherapy. A variety of factors may influence the clinical impact of any single cytotoxic agent, but drug resistance at the genetic level is thought to be one of the most important determinants in clinical outcome.

A major form of drug resistance, known as classical multidrug resistance (MDR), is characterised by cross-resistance to a variety of structurally and functionally unrelated drugs (Bradley *et al.*, 1988). The phenotype is associated with the overexpression of P-glycoprotein (P-gp), a high-molecular weight membrane protein of approximately 170 kDa (Croop *et al.*, 1988). P-gp functions as an energy-dependent drug efflux pump resulting in decreased intracellular accumulation of cytotoxic agents in resistant cells. Consequently, the regulation of P-gp expression in resistant tumour cells is the focus of many studies.

The human P-gp molecule is encoded by the *mdr*1 gene, which has been localised to chromosome 7 (Fojo, *et al.*, 1986). Chromosome 7 abnormalities have been previously described in MDR cells containing *mdr*1 gene amplification and overexpressing P-gp (Slovak *et al.*, 1987; Nieuwint *et al.*, 1992). In these cells, P-gp overexpression was commonly accompanied by various abnormalities of the long arm of chromosome 7, usually with breakpoints close to the *mdr*1 locus, 7q21.1 (Nieuwint *et al.*, 1992). However, it is currently unknown whether the structural rearrangements of chromosome 7 observed in MDR cell lines actually lead to alterations in the expression of P-gp.

It has been established that marker chromosomes can be inherited by hybrid cells resulting from somatic cell fusions (Pályi *et al.*, 1994). Therefore, the clonal chromosomal duplication of the region 7q11.23q31.2 that characterises the MDR variant of the CEM/CCRF drug-sensitive cell line, CEM/A7, (Zalcberg *et al.*, 1994) provides a marker for the investigation of the inheritance of chromosome 7 and P-gp overexpression.

In the past, studies employing somatic cell fusion techniques have been used to determine the dominance of the classical MDR phenotype. Difficulties in the interpretation of the results relate to the use of cells with complex phenotypes involving more than one mode of drug resistance and the use of a 'drug-sensitive' parental line, which in fact expressed low levels of P-gp (Eijdems *et al.*, 1992). Additionally, previous findings suggesting that the classical MDR phenotype is dominant, or at least co-dominantly expressed, have also been complicated by the use of phenotypically unstable resistant parental lines in which amplified copies of the *mdr*1 gene resided on unstable chromosomal elements (Akiyama *et al.*, 1985). In such studies, expression of *mdr*1 genes harboured on circular DNAs are not necessarily confined to the regulatory constraints of *cis*-acting factors on chromosome 7.

In the present study, although the issue of the dominance of *mdr*1 gene expression is not addressed, the expression of P-gp in hybrid cell lines was examined in a model designed to overcome the issues that confounded previous somatic cell fusion experiments. The sensitive parental line was truly P-gp negative, as determined by the polymerase chain reaction (PCR) while the resistant parental line overexpressed P-gp without the concomitant amplification of the *mdr*1 gene (Zalcberg *et al.*, 1994) seen in most classical MDR cell lines used previously. Karyotypic analyses of chromosome 7 were performed in the hybrid lines and cytotoxic agents were not added to the post fusion medium thus avoiding any selective pressure for expression of the MDR phenotype.

### Materials and methods

### *Cell lines and culture conditions*

The MDR variant of the CEM/CCRF line, CEM/A7 (Zalcberg *et al.*, 1994) was used as the drug-resistant parental line for the fusion experiments and was grown in 0.07 $\mu$g ml$^{-1}$ doxorubicin (David Bull Laboratories, Melbourne, Australia). Cells were grown in RPMI 1640 (Gibco Labs) supplemented with 10% fetal calf serum (FCS, Flow Labs, Australia) and 0.8 mM glutamine at 37°C, in a humidified chamber in an atmosphere of 5% carbon dioxide in air.

Correspondence: JR Zalcberg, Director, Medical Oncology, Austin & Repatriation Medical Centre, Repatriation Campus, Private Bag no. 1, Heidelberg W, Australia 3081
Received 7 February 1995; revised 10 July 1995; accepted 25 July 1995

The cell line, CEM/CCRF (Foley *et al.*, 1965) represented the drug-sensitive parental line into which selective markers were introduced. CEM/CCRF cells were cultured in increasing concentrations of the purine antagonist, 2-amino-6-mercaptopurine (6-thioguanine, Sigma Pharmaceuticals) and finally maintained in medium containing 20 $\mu g\ ml^{-1}$ 6-thioguanine. The resulting cell line was unable to grow in HAT medium, indicating a deficiency in the purine nucleotide salvage pathway enzyme hypoxanthine-guanine phosphoribosyl transferase (HGPRT). The resulting $HGPRT^-$/CCRF cells were stably transfected with the bacterial aminoglycoside phosphotransferase 3′ (II) gene which confers resistance to the antibiotic neomycin and its derivatives (Hanchett *et al.*, 1992). All cells were mycoplasma-free when tested by the Gen-Probe mycoplasma assay (GEN-PROBE, San Diego, CA, USA).

*Transfection of the mutant drug-sensitive parent*

$HGPRT^-$/CCRF cells, in logarithmic growth phase, were washed three times in cold, serum-free RPMI-1640 and adjusted to a concentration of $2 \times 10^7$ cells $ml^{-1}$ before electroporation. Cells (500 $\mu l$) were mixed with 20 $\mu g$ of pSV 2-neo plasmid DNA (Southern and Berg, 1982) and incubated on ice for 5 min before electroporation at 270 V and 960 $\mu F$ using a Gene Pulser (Bio-Rad Laboratories, Richmond, CA, USA). The cells were then left on ice for 10 min and resuspended in 6 ml of complete medium. The suspension was centrifuged at 800 r.p.m. for 1.5 min to remove debris and the pellet was resuspended in 10 ml of complete medium. Cells were grown in non-selective medium for 48 h, after which 400 $\mu g\ ml^{-1}$ of the neomycin analogue geneticin sulphate (G418, Gibco Laboratories, Grand Island, NY, USA) and 20 $\mu g\ ml^{-1}$ 6-thioguanine were added to the medium.

*Somatic cell fusions*

Hybrids of drug-sensitive and -resistant cells were generated by mixing $HGPRT^-/Neo^R$/CCRF (HNCCRF) sensitive cells with CEM/A7 resistant cells, in logarithmic growth phase, at a ratio of 1:5 yielding a total of $2 \times 10^7$ cells. Cells were washed three times in serum-free RPMI-1640 to remove FCS from the culture medium. Polyethylene glycol [PEG 4000, 0.8 ml, 50% w/v in phosphate-buffered saline (PBS), Boehringer Mannheim] was added dropwise over 1 min to a dispersed pellet and the suspension was incubated at 37°C for 3 min. To dilute out the PEG, 2 ml of serum-free RPMI 1640 was added over 1 min with gentle mixing. Serum-free medium (8 ml) was then added and the cell clumps were spun down at 500 r.p.m. for 3 min and resuspended in 40 ml of complete medium.

The cells were plated out into 96-well plates (Flow Labs) at a density of $5 \times 10^4$ cells per well and were incubated for 24 h in non-selective medium at 37°C. Selective medium (100 $\mu l$ of 1 × HAT: $10^{-6}$ M hypoxanthine, $4 \times 10^{-5}$ M aminopterin, $1.6 \times 10^{-5}$ M thymine from ICN Flow, California, USA; 400 $\mu g\ ml^{-1}$ G418) was added to each well. Medium was replaced with fresh selective medium every 3 days. Resultant hybrids were expanded into 24-well plates and from there into 25 $cm^2$ tissue culture flasks (Flow Labs).

*Growth assays*

Growth assays for the CEM/CCRF, HNCCRF and CEM/A7 lines were performed on actively growing cells in varying concentrations of either doxorubicin or vinblastine (David Bull Laboratories) in triplicate. The assays were performed as previously described (Hu *et al.*, 1990) with cells in logarithmic growth phase at a density of $5 \times 10^4$ cells $ml^{-1}$ seeded into 24-well plates. After 3 days, cell counts were determined using a Coulter Counter (Model DN, Coulter Electronics, Luton, England). The $IC_{50}$ value (the dose that inhibits cell growth by 50% relative to untreated controls) was determined for each cell line for both drugs. Additionally, the growth of cells in either HAT medium or varying concentrations of G418 was assessed. Cells were plated out at a density of $10^3$ cells $ml^{-1}$ and cell numbers determined following 7 and 14 days' incubation in HAT medium or 16 days' incubation in G418 at 37°C.

Similarly, drug resistance profiles for the parental CEM/CCRF, HNCCRF and CEM/A7 lines and four hybrid cell samples were determined using actively growing cells in varying concentrations of the cytotoxic agent epirubicin (David Bull Laboratories). Experiments were performed in triplicate as described above.

*MRK16 screening*

Cellular expression of P-gp was determined using a FACScan flow cytometer (Becton Dickinson, California, USA). The assay involved the use of a monoclonal antibody, MRK16 (gift from Dr Tsuruo, Division of Experimental Chemotherapy, Japanese Foundation for Cancer Research), in a method previously described (Wall *et al.*, 1993) with minor modifications. Briefly, exponentially growing cells ($10^6$ cells $ml^{-1}$) were pelleted. The pellet was dispersed and to one tube 0.37 $\mu g$ of the monoclonal antibody MRK16 was added. To the other duplicate 1 $\mu g$ of the non-specific murine monoclonal antibody, IgG 2a control (Becton Dickinson) was added to act as a control. The tubes were then incubated at room temperature for 20 min. Each sample was washed twice in complete medium and 0.5 $\mu g$ of a fluorescein-conjugated goat anti-mouse antibody (Becton Dickinson) was added. Samples were then incubated at room temperature for 20 min in the dark. Finally the cells were washed three times in complete medium and analysed in the flow cytometer.

*RNA extraction and preparation of cDNA*

RNA was extracted from both the parental cell lines and randomly selected hybrid cells using the guanidinium thiocyanate phenol chloroform method described by Chomczynski and Sacchi (1987). RNA (10 $\mu g$) and 100 ng random primers (Promega Corporation) were incubated at 70°C for 5 min and then quickly chilled on ice for 2 min. The volume was adjusted to 20 $\mu l$ containing 50 mM Tris-HCl (pH 8.3), 5 mM each of dATP, dGTP, dCTP and dTTP, 75 mM potassium chloride, 3 mM magnesium chloride, 5 mM dithiothreitol, 200 units of Superscript RNase $H^-$ Reverse Transcriptase (Gibco-BRL), and incubated at 37°C for 1 h. The resultant cDNA samples were diluted to 100 $\mu l$ with distilled water, heated to 90°C for 5 min and then stored at − 20°C.

*RT-PCR*

The primers P6 (TGCCTGGCAGCTGGAAGACAAATTCACAAAAT) and P7 (CAGACAGCAGCTGACAGTCCAAGAACAGGACTG) corresponded to nucleotides 545–577 and 799–831 relative to the *mdr*1 transcription initiation site respectively. The primers used for the histone 3.3 gene, H1 (CCACTGAACTTCTGATTCGC) and H2 (GCGTGCTAGCTGGATGTCTT), annealed to nucleotides 282–301 and 476–495 respectively. PCR reactions (50 $\mu l$) contained 25 pmol of each primer pair. Other components were 10 mM Tris-HCl, 1.5 mM magnesium chloride, 50 mM potassium chloride, 100 ng $ml^{-1}$ gelatine at pH 8.3, 100 $\mu M$ of each dNTP, 1 unit of *Taq* polymerase (Boehringer Mannheim) and 5 $\mu l$ of cDNA. The PCR cycle consisted of a single incubation at 95°C for 5 min, followed by 35 cycles of 94°C for 30 s, 58°C for 60 s and 72°C for 60 s on an Omnigene temperature cycler (Hybaid Limited, Middlesex, USA). The PCR products were analysed on 3% Nusieve GTG agarose (FMC BioProducts, Rockland, USA) gels.

*Isolation of genomic DNA and PCR*

Genomic DNA was extracted from the parental cell lines and hybrid cells according to the isolation procedure outlined by Laird *et al.* (1991). PCR followed the same protocol as above except that primers P12 (GGAAGAGCCGCTACTCGA, nucleotides 2–20) and P15 (GAAACTGTCAAGCATGCT, nucleotides 464–446) were substituted for primers P6 and P7. The PCR cycle consisted of 96°C for 2 min, 40 cycles of 95°C for 45 s, 55°C for 1 min, 72°C for 2 min.

### *Cytogenetic analysis*

Chromosome preparations were obtained using standard procedures (Webber and Garson, 1983) and G-banded chromosomes were assembled and described according to the International System of Human Cytogenetic Nomenclature recommendations (ISCN, 1991). Only chromosome 7 of each hybrid was examined in detail since the karyotype from both parental cells has been previously reported (Zalcberg *et al.*, 1994).

### Results

### *Isolation and characterisation of parental cell lines*

Two selective markers were introduced into the sensitive, parental cell line, CEM/CCRF to produce the HNCCRF cell line for use in the fusion experiments. Initially, CEM/CCRF cells were cultured in increasing concentrations of the purine antagonist 6-thioguanine to select for cells deficient in the purine nucleotide salvage pathway enzyme, HGPRT. These cells were maintained in 20 $\mu g\,ml^{-1}$ 6-thioguanine. Subsequently these HGPRT$^-$ mutant cells were electroporated with the pSVneo plasmid and cultured in medium also containing G418 (a neomycin analogue) to select for the HGPRT$^-$ and G418-resistant double mutant referred to as HNCCRF. Although a deletion or mutation in the HGPRT gene was not specifically demonstrated, the fact that this cell line was completely inhibited in HAT medium, suggests the HGPRT gene product was non-functional.

Before cell fusion, the drug resistance profile for the two parental cell lines was determined in order to see whether the creation of the double mutant, HNCCRF, had altered the drug resistance profile or induced expression of P-gp. The HNCCRF mutant remained sensitive to doxorubicin and vinblastine relative to the resistant cell line, CEM/A7. There was a minor increase in doxorubicin resistance and a decrease in vinblastine resistance relative to CEM/CCRF cells (Table I).

To confirm that HNCCRF was truly negative for P-gp expression, MRK16 binding analysis by flow cytometry was conducted. As demonstrated in Table II, P-gp expression was observed only in the CEM/A7 cell line. Both CEM/CCRF and HNCCRF failed to express P-gp. These results were confirmed by reverse transcriptase (RT)-PCR analysis of RNA extracted from these cell lines. Although all cells contained the *mdr*1 gene, no *mdr*1 mRNA was detected in the CEM/CCRF or HNCCRF lines (Table II) thus establishing HNCCRF as a P-gp-negative, drug-sensitive cell line with respect to the vinca alkaloids, drugs normally considered substrates for P-gp.

The growth of the HNCCRF cells was strongly inhibited in HAT medium but both CEM/CCRF and CEM/A7 grew well in the presence of HAT (data not shown). Conversely, G418 at concentrations as high as 800 $\mu g\,ml^{-1}$ had little effect on the survival of HNCCRF cells (data not shown). In comparison, both CEM/CCRF and CEM/A7 were incapable of growth in the presence of G418 at concentrations above 100 $\mu g\,ml^{-1}$. In summary, these data indicate that medium containing both HAT and G418 would completely inhibit the growth of the HNCCRF and CEM/A7 parental lines respectively, allowing only true hybrids to grow after somatic cell fusion.

**Table I** Drug resistance of parental cell lines to doxorubicin and vinblastine

| Drugs | CEM/CCRF | Cell lines HNCCRF | CEM/A7 |
|---|---|---|---|
| Doxorubicin | 8.6 ± 0.6 | 17.0 ± 1.0 | 357.0 ± 31.0 |
| Vinblastine | 1.9 ± 0.2 | 0.9 ± 0.1 | 37.0 ± 4.0 |

Results are expressed as the $IC_{50}$ determined as described in Materials and methods. Results represent the average concentration ($ng\,ml^{-1}$) ± standard error calculated from three identical experiments.

### *MRK16 labelling and PCR analysis of somatic cell hybrids*

Control fusions involving the crosses CEM/CCRF × CEM/A7, CEM/A7 × CEM/A7 and HNCCRF × HNCCRF yielded no viable hybrids as was expected. However, fusions between HNCCRF cells and CEM/A7 cells produced many viable hybrids.

Fourteen hybrids were randomly selected for further analysis. Preliminary analyses were carried out 5 weeks after fusion to ensure that parental cells had been eliminated by the post fusion selection medium. Ploidy analyses at this time indicated that chromosomal segregation had already taken place since most hybrids had a tetraploid complement of chromosomes, similar to that of both parental lines (data not shown). Unfortunately, most hybrids were not of sufficient confluency for analysis until 10 weeks after fusion.

Of the 14 hybrids analysed for MRK16 binding, only two were positive (1C10, 2G10). Relative to that observed in the CEM/A7 parent (100%), the binding of MRK16 in these hybrids was 86% and 75% respectively (Table II). The remainder of the hybrids were all negative for MRK16 binding, indicating no P-gp expression. The hybrids were all stable with respect to viability and MRK16 status.

RNA was extracted from 11 of the hybrids and was analysed by RT-PCR (Figure 1). The only hybrids to yield a PCR product of the expected size were 1C10 and 2G10, the same hybrids that gave a positive MRK16 labelling result. Primers to the histone 3.3 gene provided an internal control ensuring that RNA was present in each sample. Genomic DNA extracted from the same hybrids was also analysed by PCR (data not shown) using primers that anneal to the promoter region of the *mdr*1 gene. All hybrids were found to be positive for *mdr*1 genomic DNA suggesting that all hybrids had retained the *mdr*1 gene.

### *Drug resistance profile of the hybrid cells*

Resistance to epirubicin was determined in the two P-gp-positive hybrids, 1C10 and 2G10, two hybrid cell samples negative for the expression of P-gp, 2H6 and 4G9, as well as

**Table II** MRK16 binding analysis of parental and P-gp-positive hybrid cells

| Cell line | MRK16 binding[a] | MRK16 positivity[b] | RT-PCR[c] |
|---|---|---|---|
| CEM/A7 | 99 | 100 | + + + |
| CEM/CCRF | 0 | 0 | – |
| CCRF/HGPRT$^-$ | 0 | 0 | – |
| HNCCRF | 0 | 0 | – |
| 1C10 | 99 | 86 | + |
| 2G10 | 93 | 75 | + + + |

[a] Indicates the percentage of cells that were positive for MRK16 binding compared with the isotype control (see Materials and methods). [b] Positivity is the degree of MRK16 binding compared with that of the CEM/A7-positive control. [c] PCR on cDNA using primers 6 and 7 (see Materials and methods).

**Figure 1** Ethidium bromide-stained agarose gels showing the RT-PCR results from parental cell lines as well as 11 hybrids demonstrating the presence of *mdr*1 message. Histone is used as an internal control.

**Figure 2** Percentage change in the number of the parental CEM/CCRF, HNCCRF and CEM/A7 cells as well as the hybrids 1C10, 2G10, 2H6 and 4G9 as a function of increasing concentrations of epirubicin. □, CEM/CCRF; ◇, HNCCRF; O, CEM/A7; Δ, 1C10; ⊞, 2G10; ◆, 2H6; ⊕, 4G9N.

the parental cell lines CEM/CCRF, HNCCRF and CEM/A7 (Figure 2). The two hybrid cells that expressed both *mdr*l mRNA and P-gp also exhibited a resistance to the cytotoxic drug, epirubicin, similar to the MDR cell line CEM/A7. In contrast, the two drug-sensitive cell lines, CEM/CCRF and HNCCRF and the two P-gp-negative hybrids, 2H6 and 4G9, were sensitive to epirubicin.

*Cytogenetic analysis of hybrids*

The parental cell lines used in this study have been previously karyotyped (Zalcberg *et al.*, 1994). Both contain mainly near-tetraploid metaphases with the modal number of chromosomes ranging from 89 to 102. Structural changes in both cell lines are consistent as the CEM/A7 line was originally derived from the CEM/CCRF line. The main difference between the parental cell lines is that the CEM/A7 cell line contains a duplication in chromosome 7, dup(7)(q11.23q31.2). This duplication appears to include the *mdr*l gene locus which is located at 7q21.1 (Trent and Callen, 1991). Cytogenetic analysis of the hybrids revealed that only the MRK16-positive hybrids 2G10 and 1C10 contained abnormalities of chromosome 7. The specific abnormality was identified to be the same as that seen in the CEM/A7 cell line, a duplication involving the region q11.23q31.2. All other hybrids contained only normal copies of chromosome 7 as observed in the drug-sensitive parental line, CEM/CCRF. Figure 3 shows a normal chromosome 7 as well as the abnormal chromosome 7 in the hybrid 1C10.

**Figure 3** (**a**) On the left is an idiogram of a normal chromosome 7 together with a normal copy of chromosome 7 from the hybrid cell line 1C10. (**b**) On the left is an idiogram of the derivative chromosome 7 and a copy of the der(7) from 1C10 with the duplicated region 7q11.23 to 7q31.2 indicated.

### Discussion

In a previous report, we described the derivation of the drug-resistant CEM/A7 cell line in which we identified an abnormality on chromosome 7 (Zalcberg *et al.*, 1994). This chromosomal abnormality provided us with a unique marker for studying the inheritance of chromosome 7 and drug resistance in hybrids obtained from somatic cell fusion experiments.

A double mutant from the drug-sensitive human T-cell leukaemia cell line CEM/CCRF was created for use in fusion experiments. The CEM/CCRF parental cell line was selected because it lacked P-gp expression as determined by MRK16 labelling and RT-PCR analysis, thus providing a truly P-gp-negative, drug-sensitive parent. In contrast, the drug-resistant CEM/A7 cell line was strongly positive for P-gp as determined by both of these assays. The double mutant HNCCRF, was deficient in the HGPRT enzyme and carried the gene for neomycin resistance. Examination of the growth assays performed in the presence of increasing concentrations of doxorubicin and vinblastine clearly showed that HNCCRF essentially maintained its sensitivity to these drugs, having an $IC_{50}$ value similar or very close to that of the CEM/CCRF parental cell line from which it was derived (Table II) In contrast, the CEM/A7 line demonstrated resistance to both drugs, especially to doxorubicin, the drug against which it was primarily selected.

Growth of the HNCCRF and CEM/A7 cell lines was abolished in selective medium containing both HAT and G418, allowing the selection of hybrids that would have had to inherit the HGPRT enzyme from the CEM/A7 cell line and neomycin resistance from the HNCCRF cell line. This selection procedure was relatively slow, requiring about 10 weeks for most hybrids to be numerous enough for characterisation and ploidy analysis 5 weeks after fusion indicated that chromosomal segregation had already taken place (data not shown). However, compared with the cytotoxic drugs used by other groups (Ling and Baker, 1978; Eijdems *et al.*, 1992; McLean *et al.*, 1993), this selection procedure was less likely to influence the P-gp status of resulting hybrids.

Since chromosomal segregation had occurred in the hybrids, it was possible that the absence of P-gp expression in the MRK16-negative hybrids was due to the loss of the *mdr*l gene. However, PCR analysis of genomic DNA (data not shown) confirmed the presence of the *mdr*l gene in all hybrids, including those that failed to demonstrate P-gp expression. In contrast, RT-PCR analysis carried out on the same cells indicated that *mdr*l mRNA was only detectable in the two MRK16-positive hybrids, 1C10 and 2G10 (Figure 1).

The MRK16 binding results (Table II) indicate higher P-gp positivity in the 1C10 hybrid sample as compared with that of 2G10. This is supported by the functional analysis of drug resistance to the cytotoxic agent epirubicin (Figure 2) showing a higher $IC_{50}$ value for 1C10 than for 2G10 (600 ng $ml^{-1}$ and 430 ng $ml^{-1}$ respectively), suggesting that the hybrid 1C10 was more drug resistant than 2G10, at least to epirubicin. In contrast, the RT-PCR analysis (Figure 1) suggested that the expression of *mdr*1 mRNA was in fact higher in the 2G10 hybrid. However, the PCR technique used in this study was not quantitative and therefore the intensity of the bands is not indicative of the level of mRNA expression. Also, previous investigations have shown that it is possible for P-gp to be overexpressed without a simultaneous increase in *mdr*1 mRNA levels (Zhao *et al.*, 1994). Hence the RT-PCR analysis merely indicates the presence or absence of *mdr*1 mRNA.

Although the post fusion selection procedure was lengthy, preliminary characterisation of another group of hybrids during this time revealed strong MRK16 positivity. However, these hybrids were not viable at 10 weeks (data not shown). Similarly, in another group of hybrids that contained a combination of both MRK16-positive and -negative cells, only the negative cells were present 10 weeks after fusion. This apparent growth advantage of MRK16-negative cells and unstable MDR phenotype in the absence of selective pressure is not a new phenomenon. Ling and Baker (1978) added graded concentrations of colchicine to their post fusion selection medium but also noted that hybrids lost their resistance when cultured in the absence of colchicine. Akiyama *et al.* (1985) tested hybrids grown in the absence of cytotoxic agents for functional resistance at 9 and 13 weeks after fusion and demonstrated a loss of resistance. They suggested that unstable, extrachromosomal genetic elements in the parental cells may be responsible for this observation. However, in the present study the drug-resistant CEM/A7 cell line has a stable phenotype. When cultured in the absence of cytotoxic agents for more than 12 months, the cells still overexpressed P-gp, although at a reduced level (Zalcberg *et al.*, 1994).

Cytogenetic analysis of chromosome 7 in all 14 hybrid cell lines revealed the same duplication, dup(7)(q11.23q31.2) in just two hybrids, 2G10 and 1C10. This abnormality appeared to involve the *mdr*1 locus at 7q21.1. The abnormality was identical to that observed in the drug-resistant CEM/A7 parental cell line (Zalcberg *et al.*, 1994). All other hybrids contained only normal copies of chromosome 7 as seen in the drug-sensitive parental cell line, CEM/CCRF. The two hybrids with the dup(7q) karyotypic abnormality were the only two hybrids to express P-gp and *mdr*1 mRNA based on MRK16 labelling and RT-PCR assays respectively. As these two hybrids and CEM/A7 overexpress P-gp and contain the same duplication in chromosome 7, it would appear that P-gp overexpression may be linked to the inheritance of the abnormal chromosome 7 from the drug-resistant parental cell line, CEM/A7. While the inheritance of other chromosomal factors from the CEM/A7 line cannot be excluded as the cause for increased expression of P-gp, we believe the data suggest that the common karyotypic abnormalities in chromosome 7 may be the causal factor.

Although the duplicated region observed in the CEM/A7 cell line has not been characterised, it provides a marker with which to study the inheritance of chromosome 7. It is unknown whether the association shown in our data between the overexpression of P-gp and the inheritance of chromosome 7 from a resistant cell line is causally linked, however we are currently attempting to map the duplication and site of insertion of this duplication in the drug-resistant CEM/A7 cell line to further our knowledge of this chromosomal abnormality.

**Acknowledgement**
Supported in part by the Anti-Cancer Council of Victoria and the Department of Veterans Affairs, Canberra.

### References

AKIYAMA S, FOJO A, HANOVER JA, PASTAN L AND GOTTESMAN MM. (1985). Isolation and genetic characterization of human KB cell lines resistant to multiple drugs. *Somat. Cell Mol. Genet.*, **11**, 117-126.

BRADLEY G, JURANKA PF AND LING V. (1988). Mechanism of multidrug resistance. *Biochim. Biophys. Acta*, **948**, 87-128.

CHOMCZYNSKI P AND SACCHI N. (1987). Single-step method of RNA isolation by acid guanidinium thiocyanate-phenol-chloroform extraction. *Anal. Biochem.*, **162**, 156-159.

CROOP JM, GROS P AND HOUSMAN DE. (1988). Genetics of multidrug resistance. *J. Clin. Invest.*, **81**, 1303-1309.

EIJDEMS EWHM, BORST B, JONGSMA APM, DE JONG S, DE VRIES EGE, VAN GROENIGEN M, VERSANTVOORT CHM, NIEUWINT AWM AND BAAS F. (1992). Genetic transfer of non-P-glycoprotein-mediated multidrug resistance (MDR) in somatic cell fusion: Dissection of a compound MDR phenotype. *Proc. Natl Acad. Sci. USA*, **89**, 3498-3502.

FOJO AT, LEBO R, SHIMIZU N, CHIN JE, RONINSON IB, MERLINO GT, GOTTESMAN MM AND PASTAN L. (1986). Localization of MDR associated with DNA sequences to human chromosome 7. *Somat. Cell Mol. Genet.*, **12**, 415-420.

FOLEY GF, LAZARUS H, FARBER S, GEREN UZMAN B, BOONE BA AND MCCARTHY RE. (1965). Continuous culture of human lymphoblasts from peripheral blood of a child with acute leukemia. *Cancer*, **18**, 522-529.

HANCHETT LA, WANG SJ, MEEGAN RL, BAKER RM AND DOLNICK BJ. (1992). Enhanced sensitivity to G418 of human KB cells adapted to certain media and sera. *Biotechniques*, **12**, 482-486.

HU XF, MARTIN TJ, BELL DR, DE LUISE M AND ZALCBERG JR. (1990). Combined use of cyclosporin A and verapamil in modulating multidrug resistance in human leukemia cell lines. *Cancer Res.*, **50**, 2953-2957.

ISCN (1991). *Guidelines for Cancer Cytogenetics; Supplement to an International System for Human Cytogenetic Nomenclature.* Mitelman F (ed.) S Karger: Basle.

LAIRD PW, ZIJDERVELD A, LINDERS K, RUDNICKI MA, JAENISCH R AND BERNS A. (1991). Simplified mammalian isolation procedure. *Nucleic Acids Res.*, **19**, 4293.

LING V AND BAKER RM. (1978). Dominance of colchicine resistance in hybrid CHO cells. *Somat. Cell Mol. Genet.*, **4**, 193-200.

MCLEAN S, HOSKING LK AND HILL BT. (1993). Dominant expression of multiple drug resistance after *in vitro* x-irradiation exposure in intraspecific Chinese hamster ovary hybrid cells. *J. Natl Cancer Inst.*, **85**, 48-53.

NIEUWINT AWM, BAAS F, WIEGANT J AND JOENJE H. (1992). Cytogenetic alterations associated with P-glycoprotein and non-P-glycoprotein-mediated multidrug resistance in SW-1573 human lung tumour cells lines. *Cancer Res.*, **52**, 4361-4371.

PÁLYI I, TURI G, HULLÁN L, SZIKLA K AND BAK M. (1994). Dominant expression of multidrug resistance in intraspecific murine lymphoma hybrid cells. *Cancer Chemother. Pharmacol.*, **34**, 81-85.

SLOVAK ML, HOELTGE GA AND TRENT JM. (1987). Cytogenetic alterations associated with the acquisition of doxorubicin resistance: possible significance of chromosome 7 alterations. *Cancer Res.*, **47**, 6646-6652.

SOUTHERN PJ AND BERG P. (1982). Transformation of mammalian cells to antibiotic resistance with a bacterial gene under control of the SV40 early region promoter. *J. Mol. Appl. Genet.*, **1**, 327-341.

TRENT JM AND CALLEN DF. (1991). Chromosome localization of P-glycoprotein genes in drug-sensitive and -resistant human cells. In *Molecular and Cellular Biology of Multidrug Resistance in Tumor Cells*, Roninson IB (ed.) pp. 169-188. Plenum Press: New York.

WALL DM, SPARROW R, HU XF, NADALIN G, ZALCBERG JR, VAN DER WEYDEN M AND PARKIN JD. (1993). Clinical application of a rapid, functional assay for multidrug resistance based on accumulation of the fluorescent dye, fluo-3. *Eur. J. Cancer*, **29A**, 1024-1027.

WEBBER LM AND GARSON OM. (1983). Flurodeoxyruidine synchronization of bone marrow cultures. *Cancer Genet. Cytogenet.*, **26**, 123–132.

ZALCBERG JR, HU XF, WALL DMP, MIRSKI S, COLE S, NADALIN G, DE LUISE M, PARKIN JD, VRAZAS V, CAMPBELL L AND KANTHARIDIS P. (1994). Cellular and karyotypic characterization of two doxorubicin resistant cell lines isolated from the same parental human leukemia cell line. *Int. J. Cancer*, **57**, 522–528.

ZHAO J-Y, SAVARAJ N, SONG R, PRIEBE W AND KUO MT. (1994). Overexpression of P-glycoprotein but not its mRNA in multidrug resistant cells selected with hydroxyrubicin. *Anticancer Res.*, **14**, 1735–1742.